# Supplementary material for: Investigating the Effects of a New Peptide, Derived from the Enterolobium contortisiliquum Proteinase Inhibitor (EcTI), on Inflammation, Remodeling, and Oxidative Stress in an Experimental Mouse Model of Asthma–Chronic Obstructive Pulmonary Disease Overlap (ACO)
Source: Int J Mol Sci. 2023 Sep 28;24(19):14710. doi: 10.3390/ijms241914710 (PMC10573003; doi:10.3390/ijms241914710)
Supplement: Supplementary file 1 [file ijms-24-14710-s001.zip › ijms-2612619-supplementary.pdf]

Supplementary materials:

	SAL	SAL-PEP-EcTI	*p* Value
**Airway Hyperresponsiveness to Methacholine**			
**%Rrs**	73.50±8.91	106.84±22.61	p=0.153
**%Ers**	77.30±7.56	18.50±3.20	p=<0.001
**%Gtis**	35.45±6.59	45.77±6.66	p=0.297
**%Htis**	46.99±2.63	16.54±2.87	p=<0.001
**%Raw**	256.34±36.86	355.75±82.47	p=0.293
**BALF (cells/10^4 µm^2)**			
**Total cells**	0.70±0.12	0.53±0.06	p=0.248
**Eosinófilos**	0.17±0.04	0.10±0.02	p=0.198
**Neutrófilos**	0.16±0.03	0.10±0.02	p=0.186
**Linfócitos**	0.18±0.03	0.20±0.03	p=0.699
**Macrófagos**	0.19±0.03	0.11±0.03	p=0.183
**Marcadores inflamatórios (células/10^4 µm^2)**			
**IL-1-β - Vias aéreas**	0.41±0.12	0.75±0.15	p=0.088
**IL-1-β - Paredes Alveolares**	0.47±0.17	0.96±0.20	p=0.109
**IL-5 - Vias aéreas**	1.63±0.24	1.69±0.30	p=0.909
**IL-5 - Paredes Alveolares**	1.25±0.19	1.33±0.21	p=0.780
**IL-13 - Vias aéreas**	2.76±0.34	3.35±2.98	p=0.280
**IL-13 - Paredes Alveolares**	3.18±0.43	2.92±0.38	p=0.687
**TNF-α - Vias aéreas**	1.82±0.40	1.21±0.51	p=0.695
**INF-γ - Vias aéreas**	1.02±0.19	1.21±0.51	p=0.807
**Remodelamento (células/10^4 µm^2)**			
**MMP-12 - Vias aéreas**	1.98±0.37	1.60±0.29	p=0.522
**MMP-12 - Paredes Alveolares**	0.66±0.19	1.19±0.24	p=0.089
**Estresse oxidativo (células/10^4 µm^2)**			
**iNOS - Vias aéreas**	3.34±0.59	3.06±0.94	p=0.791
**iNOS - Alveolar septa**	2.17±0.30	1.79±0.23	p=0.315
**Fator de transcrição (células/10^4µm^2)**			
**NF-κB - Vias aéreas**	0.57±0.19	1.06±0.19	p=0.076
**NF-κB - Paredes Alveolares**	0.34±0.18	0.83±0.19	p=0.69
